# On triatomines, cockroaches and haemolymphagy under laboratory conditions: new discoveries

**DOI:** 10.1590/0074-02760160027

**Published:** 2016-10-03

**Authors:** Pamela Durán, Edda Siñani, Stéphanie Depickère

**Affiliations:** 1 Universidad Mayor de San Andrés, Instituto de Investigación en Salud y Desarrollo, Cátedra de Parasitología, La Paz, Bolivia; 2 Instituto Nacional de Laboratorios de Salud, Laboratorio de Entomología Médica, La Paz, Bolivia; 3 Institut de Recherche pour le Développement, Embajada Francia, La Paz, Plurinational State of Bolivia

**Keywords:** Triatominae, haemolymphagy, survival, fecundity, life cycle, Bolivia

## Abstract

For a long time, haematophagy was considered an obligate condition for triatomines (Hemiptera: Reduviidae) to complete their life cycle. Today, the ability to use haemolymphagy is suggested to represent an important survival strategy for some species, especially those in genus *Belminus*. As *Eratyrus mucronatus* and *Triatoma boliviana* are found with cockroaches in the Blaberinae subfamily in Bolivia, their developmental cycle from egg to adult under a “cockroach diet” was studied. The results suggested that having only cockroach haemolymph as a food source compromised development cycle completion in both species. Compared to a “mouse diet”, the cockroach diet increased: (i) the mortality at each nymphal instar; (ii) the number of feedings needed to molt; (iii) the volume of the maximum food intake; and (iv) the time needed to molt. In conclusion, haemolymph could effectively support survival in the field in both species. Nevertheless, under laboratory conditions, the use of haemolymphagy as a survival strategy in the first developmental stages of these species was not supported, as their mortality was very high. Finally, when *Triatoma infestans*, *Rhodnius stali* and *Panstrongylus rufotuberculatus* species were reared on a cockroach diet under similar conditions, all died rather than feeding on cockroaches. These results are discussed in the context of the ecology of each species.

Triatomines (Hemiptera: Reduviidae) are also known as kissing bugs. Approximately 150 species have been described; all of them are haematophagous and considered as potential vectors of *Trypanosoma cruzi* (Kinetoplastida: Trypanosomatidae), the aetiological agent of Chagas disease or American trypanosomiasis (Telleria & Tibayrenc 2010, Justi et al. 2014). Chagas disease is endemic to the Americas and represents a major sanitation issue in Central and South America (WHO 2010). Most triatomine species are sylvatic and feed on small terrestrial and arboreal mammals, especially didelphids, edentates, and rodents, or live in association with bats or birds. Only a few species have colonised human dwellings, where they can transmit the *T. cruzi* parasite to humans and domestic mammals (Lent & Wygodzinsky 1979, Carcavallo et al. 1998, Telleria & Tibayrenc 2010).

Although feeding on invertebrates has been observed in some species (Brumpt 1914, Ryckman 1951, Miles et al. 1981), haematophagy was historically considered an obligate condition for triatomines to complete their life cycle (Lent & Wygodzinsky 1979). Haematophagy is the most common feeding practice in the triatomine subfamily; nevertheless, three other practices have been described: cleptohaematophagy, or feeding on the ingested blood meal of another triatomine; haemolymphagy, or feeding on arthropod haemolymph (Sandoval et al. 2010); and phytophagy, or feeding on sugar meal, which was recently demonstrated (Díaz-Albiter et al. 2016). In haemolymphagy, three different behaviours can be distinguished: intraspecific haemolymphagy (also called cannibalism by some authors and defined as a feeding on the haemolymph from individuals of the same species); intrasubfamily haemolymphagy (also called ‘ectoparasite’ cannibalism and defined as feeding from an individual of another Triatominae species); and extrasubfamily haemolymphagy, defined as feeding on haemolymph from a non-triatomine invertebrate (Sandoval et al. 2010).

With the increase in the literature regarding the feeding sources of triatomines over the last 30 years, haemolymphagy has been observed in more than 20 species belonging to the genera *Belminus* (Lent & Wygodzinsky 1979, Sandoval et al. 2000, 2004, 2010, 2013), *Eratyrus* (Miles et al. 1981), *Psammolestes* (Carcavallo et al. 1998, Noireau et al. 2005a), *Panstrongylus* (Carcavallo et al. 1998, Garrouste 2009), *Rhodnius* (Brumpt 1914, Marinkelle 1965, Piñero et al. 1988) and *Triatoma* (Brumpt 1914, Ryckman 1951, Phillips 1960, Lent & Wygodzinsky 1979, Salvatella & Calegari 1991, Salvatella et al. 1994, Lorosa et al. 2000, Ruas-Neto et al. 2001, Vezzani et al. 2001, Emmanuelle-Machado et al. 2002, Freitas et al. 2005, Noireau et al. 2005a, Silva et al. 2005, Zeledón et al. 2010, Alves et al. 2011, Pontes et al. 2011, Cardozo-de-Almeida et al. 2014). The ability to use haemolymphagy is suggested to represent an important survival strategy under natural conditions for some species, resulting in optimised use of available nutrition resources and an increase in the probability of survival when vertebrate hosts are absent (Salvatella et al. 1994, Lorosa et al. 2000, Ruas-Neto et al. 2001, Alves et al. 2011). Complete egg-to-adult development seems to be seriously compromised by a diet of haemolymph alone, except in species within the genus *Belminus*, in which haemolymphagy seems to predominate and vertebrate blood is an infrequent food source (Sandoval et al. 2010, 2013).

In Bolivia, two species of triatomines found inside and/or around human dwellings, *Eratyrus mucronatus* and *Triatoma boliviana*, were also frequently found in the presence of large cockroaches of the *Blaberus* sp. (8 cm long for adults) in the subfamily Blaberinae (Martínez et al. 2007, Depickère et al. 2012, Durán et al. 2014). Interestingly, both species naturally demonstrate a null or weak level of infection by *T. cruzi* [proportion of infected insects: *E. mucronatus*: 0% by microscopical observation of 68 faeces (Noireau et al. 1995); 19.1% by polymerase chain reaction (PCR) test of 68 faeces (Noireau et al. 1995); 0% by microscopical observation of 75 faeces (Depickère et al. 2012); *T. boliviana*: 0% by microscopical observation of 325 faeces (Depickère, pers. comm.)]. *E. mucronatus* is found in dwellings in a region of the Department of La Paz, in Bolivia (Depickère et al. 2012). Colonies of this species were observed in the boundary walls of peridomiciles constructed of tapial, or rammed earth. The tops of these walls were covered by branches and earth and accommodated triatomines, cockroaches, and other invertebrates. Haemolymphagy on spiders has been previously described in this species (Miles et al. 1981). In the laboratory, they feed readily on hens and mice. *T. boliviana* is found in another region of the Department of La Paz in stone walls delimiting fields (Martínez et al. 2007, Durán et al. 2014). Little is known about their natural food sources. In the laboratory, they will feed on mice, but not on hens.

A laboratory study was performed to verify whether these two species were able to feed on the haemolymph of cockroach species found within their capture zones and complete their life cycle with only this food source. Other triatomine species found in Bolivia (*Triatoma infestans*, *Rhodnius stali* and *Panstrongylus rufotuberculatus*) were also provided the same “cockroach diet” as a preliminary investigation of their ability to exploit this food source, though there have not yet been any reports indicating that these species live in association with cockroaches.

## MATERIALS AND METHODS

Species were reared under controlled laboratory conditions: *E. mucronatus* (captured in Province of Franz Tamayo, La Paz), *T. infestans* (Province of Oropeza, Chuquisaca), *P. rufotuberculatus* (Province of Muñecas, La Paz) and *R. stali* (Province of Sud Yungas, La Paz) were kept under 26 ± 2ºC and 60 ± 10% RH; *T. boliviana* (Province of Muñecas, La Paz) was kept under 22 ± 2ºC and 60 ± 10% RH. The dark:light cycle was 12:12 for all species. The study began with first instar nymphs (N1) from the first generation (F1) as soon as they hatched. The development of the cohort was followed through the nymphal instar and adult stages until death. Each nymph was reared individually. Adults were reared in pairs to observe fecundity and then egg viability. Insects had the opportunity to feed for two hours, twice a week. Two hours has been observed as an adequate duration of feeding ad libitum for triatomines. The food intake was determined by the increase in weight, as measured on a precision balance (Precisa Instrument Switzerland, XT220A). The life-time, mortality rate and number of feedings were recorded for each instar. The results were compared with those obtained in the same conditions but using a mouse as the food source. Due to the small number of individuals, data were analysed with non-parametric tests (Mann-Whitney U test, Kruskal-Wallis test, Chi-squared test and Fisher Exact test) using R (R team).

*Diet* - *Blaberus* sp. cockroaches (Blattodea: Blaberidae: Blaberinae, Philippe Grandcolas, MNHN Paris, pers. com.) were captured at the same time as the triatomines*.* Cockroaches were reared under laboratory conditions and supplied with food (laboratory rodent food) and water ad libitum. A total of 37 N1 *E. mucronatus* and 351 N1 *T. boliviana* were used in the study. Nymphs of *E. mucronatus* were fed only on cockroaches until their death. For *T. boliviana*, in light of the high mortality of the N1*,* insects were reared on different diets to detect if they could exploit haemolymphagy as a survival strategy (see Table I for details). Insects were divided into four groups according to their diet: (i) 100% of feedings on cockroach haemolymph (Cc); (ii) 100% of feedings with a choice between cockroach and mouse (Ch); (iii) N1 on mouse, and then from N2 until death on cockroach (MCc); and finally (iv) N1 on cockroach, N2 and N3 with a choice of mouse or cockroach, and from N4 until death only on cockroach (CcChCc). Finally, five N1 of *T. infestans*, 18 N1 of *P. rufotuberculatus*, 32 N1 and 15 adults of *R. stali* were also provided a cockroach diet under the conditions previously described.

Results were compared with those obtained for insects reared under the same conditions of temperature and humidity but feeding on mice throughout their life cycle (mouse diet, abbreviated: M).

*Feeding setup* - A triatomine was placed in a small jar of 7 cm diameter and 8 cm height, closed with a piece of tulle held in place by a rubber band. A piece of paper folded into pleats was introduced vertically into the jar to link the bottom of the jar and the tulle and offer a vertical resting place for the insect. In the case of feeding on a cockroach, the cockroach (adult or N5, 5-8 cm long) was introduced into the jar with the triatomine. In the case of feeding on a mouse, the mouse was placed inside a thin wire mesh tube that did not injure the mouse but prevented movement (similar to the setup used in Klotz et al. 2009); the immobilised mouse was then placed on the tulle piece. The bug was able to feed by climbing on the paper piece and ingesting blood through the tulle. In the case of a choice in food source, both mouse and cockroach were offered to the triatomine.

*Effect of cockroach diet on infected triatomines* - As explained previously, *E. mucronatus* and *T. boliviana* in Bolivia have a very low infection index, with parasites being detected only by PCR. The hypothesis that a haemolymph diet could decrease the *T. cruzi* infection rate in triatomines was tested in both species by a small experiment: triatomines (one ♀ *E. mucronatus*, one ♀, five ♂ and three N5 of *T. boliviana*) were artificially infected with a strain of *T. cruzi* extracted from infected *T. infestans* specimens captured in Province of Murillo, La Paz Department. These experimentally infected triatomines were then provided a cockroach diet (twice a week, same conditions of rearing and feeding as described above), and the infection rate of the triatomines was obtained at 49 and 70 days by observation of the faeces (obtained by gently squeezing live triatomines, mixing the faeces with physiological serum and analysing the mixture using a light microscope to identify the presence of flagellates).

*Ethics* - The experiments were performed at the National Institute of Laboratories in Health (INLASA), which reports to the Health Ministry and operates according to the national law on the care and use of laboratory animals. Mice were obtained from the Rabies Vaccine Production Laboratory of INLASA’s mouse husbandry lab. Particular attention was taken to ensure animal welfare in our experiments (use of one mouse to feed only one insect every two weeks; gentle immobilisation of the mouse during triatomine feeding). All the experiments were approved by the Institute.

## RESULTS

Triatomines could ingest haemolymph from different physiological parts of the cockroach (ventral part, legs, head); the most frequent feeding location was the legs (Figure).

**Figure:**
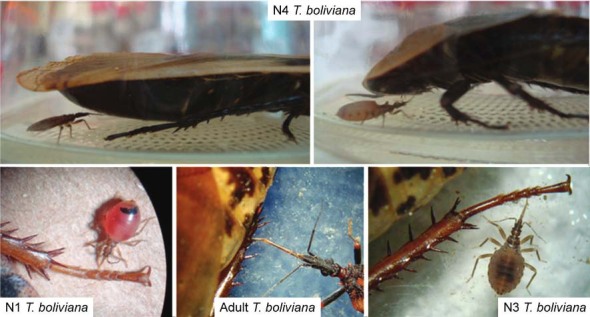
*Triatoma boliviana* feeding on cockroach haemolymph. They could ingest haemolymph from different parts of the cockroach (ventral part, legs, head), with the most frequent location being the legs.

*E. mucronatus* - Of the 37 N1 in the experiment, only one specimen developed into an adult (Table I). The mortality rate was high in each instar (> 50%). On a mouse diet, the mortality rate was also increased, especially for N1 and N5 (Table I), but it was significantly lower than the mortality rate of triatomines on a cockroach diet (Fisher Exact test: p = 0.002). Analysis of N1 mortality showed that the nymphal mortality occurred (i) before a successful feeding, (ii) before molting after feeding at least one time, or (iii) during molting. The greatest difference between diets was in the proportion of nymphs that fed and died before molting (Table II). Indeed, the latter was higher on a cockroach diet (46%) than on a mouse diet (14%). This type of death occurred at an age that was not significantly different from the age of molting in both diets (Mann-Whitney U test comparing the lifetimes of N1 that molted and those that died before molting: cockroach diet: W = 94, p = 0.50; mouse diet: W = 28, p = 0.52). N1 that ate and died before molting had a significantly smaller weight than those that succeeded to molt on the cockroach diet (Mann-Whitney U test: W = 53, p = 0.016). No difference was observed between these two groups on a mouse diet (Mann-Whitney U test: W = 19.5, p = 0.17). The day of the first meal did not differ between the cockroach and a mouse diets (Mann-Whitney U test: W = 77, N_Cc_ = 13, N_M_ = 18, p = 0.11).

**TABLE I:** Number of insects and percent mortality of *Eratyrus ucronatus* and *Triatoma boliviana* present at each instar, for all diet types

	*E. mucronatus*		*T. boliviana*				
		
	Cockroach	Mouse	Cc	CcChCc	Ch	MCc	Mouse
N1	37 (65)	29 (41)	179 (93)		44 (91)	-	95 (67)
N2	13 (61)	17 (18)	5 (60)	7 (43)	4 (75)	33 (67)	31 (6)
N3	5 (60)	14 (7)	2 (50)	4 (25)	1 (100)	11 (73)	29 (0)
N4	2 (0)	13 (8)	1 (0)	3 (67)	0	3 (67)	29 (3)
N5	2 (50)	12 (58)	1 (100)	1 (0)	0	1 (0)	28 (18)
Adults	1♂	5 (4♂, 1♀)	0	1♂	0	1♀	23(8♂, 15♀)

Cc (100% ockroach); CcChCc (N1, N4 and N5 on cockroach and choice cockroach/mouse for N2 and N3); Ch (100% choice mouse/cockroach); MCc (N1 on mouse, and then N2 to N5 on cockroach) and M (100% mouse).

**TABLE II:** *Eratyrus mucronatus* and *Triatoma boliviana* N1 development on the different diets types

	*E. mucronatus* (N1)		*T. boliviana* (N1)		
		
	Cockroach	Mouse	Cockroach	Choice	Mouse
No eating	5 (14)	4 (14)	106 (59)	24 (55)	63 (66)
Eating but no molt	17 (46)	4 (14)	61 (34)	16 (36)	1 (1)
Eating, death during molting	2 (5)	3 (10)	-	-	-
Eating and molt	13 (35)	18 (62)	12 (7)	4 (9)	31 (33)

Number (%) of N1 that: (i) did not eat and died; (ii) ate and died before molting; (iii) ate, but died during molting; and (iv) ate and molted.

Triatomines reared on cockroach needed a higher number of meals to molt, especially for N1, N2 and N3 (Table III). Comparing the maximum gain of weight per instar between the cockroach and mouse diets, the nymphal instars reared on cockroach tended to have a higher weight increase, except for N1, in which food intake was significantly lower on the cockroach diet (Table III). The age of molting tended to be higher on the cockroach diet (Table III).

**TABLE III:** Development of nymphal instars of *Eratyrus mucronatus*

	Feeding number			Weight max / weight at molting			Molting age (days)		
			
	Cockroach	Mouse	W, p-value	Cockroach	Mouse	W, p-value	Cockroach	Mouse	W, p-value
N1	4.0 (3.0-5.0)	1.5 (1.0-2.0)	229.5, < 0.001	6.3 (5.6-7.7)	8.4 (8.0-9.5)	46.5, < 0.01	36 (34-40)	33 (29-37)	164, 0.06
N2	3.0 (2.0-5.0)	1.0 (1.0-2.0)	61, 0.01	6.9 (6.4-7.0)	4.6 (3.7-4.8)	69, < 0.001	35 (35-42)	26 (24-28)	61, 0.01
N3	4.0 (4.0-4.0)	2.0 (1.0-2.0)	26, 0.02	5.7 (4.6-6.7)	4.8 (4.3-4.9)	14, 0.93	42 (38-45)	28 (25-33)	22.5, 0.13
N4	3.5 (2.8-4.3)	2.0 (2.0-2.0)	18, 0.24	4.8 (4.1-5.5)	4.1 (3.9-5.1)	11, 0.92	51 (50-51)	31 (28-36)	24, 0.03
N5	7.0 (-)	6.0 (5.0-6.0)	4, 0.55	3.9 (-)	2.9 (2.6-3.4)	5, 0.33	99 (-)	60 (49-66)	5, 0.33

The number of feedings, maximum increase in weight (expressed as the maximum weight achieved by the nymph at instar *i* divided by its weight just after molting into instar *i*) and molting age for each instar and for both a cockroach and a mouse diets are provided. All data are expressed in median values with interquartile ranges. The W and p-value for the Mann-Whitney U test to compare data between the diets is provided.

Only one specimen reached the adult stage (male) on a cockroach diet. It lived 276 days, which is older than the median life expectancy of males on a mouse diet (134 days, max = 265 days, min = 80 days, 12 males observed). Its frequency of cockroach feeding was once per eight days, which is more frequently than was observed on a mouse diet (median feed frequency: 12 days, max = 21 days, min = 10 days, 12 males observed). Its median rate of weight increase per feeding was 1.1, which is similar to the rate observed for males on a mouse diet (median rate of 1.1, max = 3.5, min = 0.4, 12 males observed). To observe its fecundity, a female (fed on mouse) that had recently emerged was added; ten eggs were laid, from which two hatched and survived. The female died after two months.

*T. boliviana* - The proportion of nymphs reaching the adult stage was zero for insects rearing on the Cc or Ch diets and very low among those reared on the CcChCc (one male) or MCc diets (one female, Table I). In contrast, 24% of the N1 instars developed into adults on the mouse diet.

The N1 mortality rate was significantly lower for nymphs fed on mouse than on cockroach or having the choice between mice or cockroach feedings (Table I, respectively 67%, 93% and 91%, Chi-squared test: X^2^ = 34.4, p < 0.001; Fisher Exact test: p < 0.001). Triatomines from first nymphal instar died without feeding (while the possibility of feeding was given) or died before molting. No significant difference was found in the proportion of N1 that died without feeding between the three diets (Table II, Chi-squared test: X^2^= 2.1, p = 0.35; Fisher Exact test: p = 0.34). The proportion of nymphs that fed and died before molting was similar between the cockroach diet and choice diet; this proportion was higher than in the group fed the mouse diet (Chi-squared test: X^2^= 40.4, p < 0.001; Fisher Exact test: p < 0.001). The N1 age at the first meal intake did not differ between the mouse and a cockroach diets (Mann-Whitney U test: W = 130.5, N_Cc_ = 12, N_M_ = 27, p = 0.33).

The number of feedings and the maximum food intake for N1 instars were not significantly different between the three diets (Table IV, Kruskal-Wallis test: X^2^ = 5.7, p = 0.06; and X^2^ = 1.2, p = 0.57, respectively). The time for molting was significantly higher for triatomines on the choice diet than for those on a mouse diet (Kruskal-Wallis test: X^2^ = 8.8, p = 0.01 followed by a Bonferroni multiple comparisons test). When N1 molted to N2, the weight of emerging nymphs reared on the cockroach and choice diets were statistically significantly lower than nymphs on the mouse diet (Kruskal-Wallis test: X^2^ = 13.8, p = 0.001 followed by a Bonferroni multiple comparison test). Even if low survival rates prevented the statistically significant determination of the impact of the diets on the development, a trend was clearly observable: the cockroach diet tended to increase the maximum food intake and number of feedings required for molting, resulting in an increased molting period (almost twice that of mouse-diet fed triatomines) and a smaller weight at emergence. When choice between feeding on a mouse or cockroach was provided, nymphs N2 and N3 tended to prefer to feed on a mouse (24 feedings observed from six N2 and three N3: 17 feedings on mouse and seven on cockroach).

**TABLE IV:** Development of nymphal instars of *Triatoma boliviana*

Diet	Cc	CcChCc	Ch	MCc	M
Feeding number (Q1-Q3), *Number of studied insects*					

N1	3.0 (2.0-3.2), *12*		2.5 (2.0-3.0), *4*	-	2.0 (2.0-2.5), *31*
N2	3.0 (3.0-3.0), *2*	2.5 (1.8-3.0), *4*	4.0 (-), *1*	4.0 (3.0-5.5), *11*	2.0 (1.0-2.0), *29*
N3	5.0 (-), *1*	2.0 (2.0-2.5), *3*	-	4.0 (4.0-6.0), *3*	2.0 (2.0-3.0), *29*
N4	2.0 (-), *1*	5.0 (-), *1*	-	9.0 (-), *1*	4.0 (3.0-4.0), *28*
N5	-	7.0 (-), *1*	-	11 (-), *1*	6.0 (5.0-7.0), *23*

Weight max / weight at molting					

N1	7.3 (6.1-8.7), *12*		7.6 (7.2-8.4), *4*	-	7.0 (6.1-7.9), *31*
N2	6.6 (6.6-6.6), *2*	7.1 (6.9-7.2), *4*	8.8 (-), *1*	4.8 (4.1-6.4), *11*	5.8 (5.0-7.3), *29*
N3	4.3 (-), *1*	4.8 (4.3-5.0), *3*	-	4.8 (4.7-5.5), *3*	4.7 (4.2-5.2), *29*
N4	6.5 (-), *1*	4.2 (-), *1*	-	6.9 (-), *1*	3.9 (3.5-4.2), *28*
N5	-	4.1 (-), *1*	-	6.6 (-), *1*	3.1 (2.7-3.3), *23*

Molting age (days)					

N1	42 (37-43), *12*		56 (52-58), *4*	-	35 (34-41), *31*
N2	49 (44-55), *2*	31 (30-33), *4*	28 (-), *1*	62 (49-70), *11*	28 (28-32), *29*
N3	60 (-), *1*	31 (30-38), *3*	-	50 (45-68), *3*	34 (31-35), *29*
N4	73 (-), *1*	84 (-), *1*	-	90 (-), *1*	45 (39-46), *28*
N5	-	98 (-), *1*	-	140 (-), *1*	70 (64-83), *23*

Weight N_i_ at molting / Weight N_i-1_ at molting					

N1	3.1 (2.9-3.4), *12*		2.4 (2.0-2.8), *4*	-	4.5 (3.5-5.0), *31*
N2	3.6 (2.6-4.5), *2*	4.0 (3.8-4.3), *4*	5.3 (-), *1*	1.7 (1.6-2.4), *11*	3.8 (3.0-4.3), *29*
N3	2.2 (-), *1*	2.6 (2.3-3.0), *3*	-	2.4 (1.9-2.8), *3*	3.0 (2.9-3.6), *29*
N4	1.6 (-), *1*	1.5 (-), *1*	-	2.9 (-), *1*	2.5 (2.4-2.6), *28*
N5	-	1.9 (-), *1*	-	1.9 (-), *1*	1.9 (1.6-2.0), *23*

The number of feedings, maximum increase in weight (expressed as the maximum weight achieved by the nymph at instar *i* divided by its weight just after molting into instar *i*), molting age, and weight increase from one instar to the next one (expressed as the weight of N_i_ at molting divided by the weight of N_i-1_ at molting) are given for each instar and for all diet type. All data are expressed as median values with interquartile ranges; the number of studied insects is provided in italic. Cc (100% cockroach), CcChCc (N1, N4 and N5 on cockroach and choice cockroach /mouse for N2 and N3), Ch (100% choice mouse/cockroach), MCc (N1 on mouse, and then N2 to N5 on cockroach) and M (100% mouse).

Two nymphs reached adulthood: one female (MCc diet) and one male (CcChCc diet). The weight of these adults after molting was lower than the median weight of insects fed only on mouse [female: 0.0803 g, median and interquartiles of the weight on the mouse diet: 0.1429 g (0.1269-0.1578); male: 0.0807 g, 0.1182 g (0.1048-0.1345)]. Compared with insects reared on the mouse diet, adults feeding on cockroach seemed to have a higher, but not more frequent, food intake (Table V). The lifespan of this female was lower than the median lifespan of the insects fed on mouse; in contrast, the lifespan of the male was higher (Table V). The female on the MCc diet was only fed on cockroach and reared in pair with a male fed on mouse. This female did not lay eggs. The male on the CcChCc diet was also only fed cockroach. It was reared with three females on the mouse diet. After their emergence, two of these females (A and B) were offered only cockroaches, and one was offered only mice (C, Table V). For females A and B, one did not lay any eggs; the other female laid 13 eggs, of which six N1 hatched and survived (median hatching time: 38 days). The female C laid 28 eggs, of which only one N1 hatched and survived (hatching time: 39 days).

**TABLE V:** Comparison of feeding behaviour and fecundity among adults of *Triatoma boliviana* under cockroach and mouse diets

	Lifetime (days)	Time between feeding (days)	Ratio of weigh after feeding	Number of hatched eggs (total number)
Female (MCc) *n* = 1	81	4 (3-7), *a* = 13	2.0 (1.9-2.1), *a* = 13	0 (0)
Females (M) *n* = 14	235 (184-286)	6 (4-7), *a* = 512	1.0 (1.0-1.1), *a* = 512	732 (1448)
MW U-test	-	W = 2969, p = 0.50	**W = 5, p < 0.001**	

Male (CcChCc) *n* = 1	417	5 (3-10), *a* = 59	1.2 (1.0-1.3), *a* = 59	-
Males (M) *n* = 8	140 (123-156)	7 (4-7), *a* = 168	0.9 (0.9-1.0), *a* = 158	-
MW U-test	-	W = 4914.5, p = 0.92	**W = 1635, p < 0.001**	

Female A (Cc)	161	9 (3-13)	1.4 (1.0-1.5)	6 (13)
Female B (Cc)	145	5 (3-9)	0.9 (0.8-0.9)	0 (0)
Female C (M)	280	7 (4-10)	1.6 (1.5-1.7)	1 (28)

Lifetime (days), time between two feedings (days), increase of weight after feeding and the number of hatched eggs are provided. All data are expressed as median values with interquartile ranges. The p-value for the Mann-Whitney U test to compare data between the diets is provided. *n*: the number of observed triatomines; *a*: is the number of feeding observations. The feeding diet of study insects is also provided (MCc, M, CcChCc, see Materials and Methods section 2.1 for more information).

*T. infestans, P. rufotuberculatus* and *R. stali* - As few insects and instars were included from these three species, these results must be considered preliminary observations of other species under the same conditions. Concerning *T. infestans*, none of the N1 fed on cockroach. Mortality occurred at the age of 67-86 days and 16-22 contacts with the cockroach. All N1 of *P. rufotuberculatus* died without feeding at a median age of 12 days old after two to three contacts with the cockroach. Only one nymph succeeded once in feeding on a small amount of cockroach’s haemolymph, but it died 10 days later. For the 32 N1 of *R. stali*, none fed on cockroach. They died between eight-47 days old (median: 13), after one-12 contacts with the cockroach (median: two). None of the adults of *R. stali* fed on the cockroach. Males survived the starvation for somewhat longer than females (58 versus 40 days, respectively; Mann-Whitney U test: N♂ = 7, N♀ = 8, W = 10.5, p = 0.048) and had a higher number of contacts with the cockroach (median number of contacts for males: 15, and for females: 10).

*Effect of cockroach diet on infected triatomines* - Moving parasites were detected by microscopy in all the triatomines of both species at 49 and 70 days (triatomines fed ~once a week on cockroach haemolymph).

## DISCUSSION

Haemolymphagy could be a good strategy for triatomines to survive in a wild environment offering few vertebrate hosts. Some species have already been reported to be able to exploit haemolymph to survive (Alves et al. 2011, Pontes et al. 2011) or even to use it as a principal food source (Sandoval et al. 2010, 2013). Cockroaches are present in a variety of habitats and could represent a source of haemolymph with relatively easy access. Moreover, they are the principal food source of the *Belminus ferroae* triatomine species (Sandoval et al. 2010, 2013). Nevertheless, the results presented here suggest that haemolymphagy using adult/N5 cockroaches as a food source is uncommon in the studied species.

Although the results for *T. infestans, P. rufotuberculatus* and *R. stali* must be considered as preliminary and studied further, data from these species can be discussed here to orient future research. *T. infestans* N1 died rather than feed on cockroaches. Their resistance to starvation was very high compared with the other species (67-86 days). Some wild foci have been identified (Noireau et al. 2005b, Bacigalupo et al. 2010, Buitrago et al. 2010, Waleckx et al. 2012), but they generally live in large numbers inside or around the human dwellings where vertebrate hosts are continuously present. Recently, Alves et al. (2011) found that nymphal instars of *T. infestans* are able to practice intrasubfamily haemolymphagy and cleptohaematophagy. In the case of a lack of vertebrate hosts, this species might be more inclined to practice cleptohaematophagy/intrasubfamily haemolymphagy than to feed on haemolymph from other arthropods such as cockroaches. *P. rufotuberculatus* refused also to feed on cockroach under our conditions. Just one first instar fed once on haemolymph, but it was not sufficient to survive. Their resistance to starvation was low under our conditions (12 days). In Bolivia, this species lives in human dwellings in association with guinea pigs reared by inhabitants, which are also a stable food source (Depickère et al. 2011). Few data are available regarding their natural food sources in the wild; however, they are reported to feed on Dasypodidae, Procyonidae and bats (Carcavallo et al. 1998). In the genus *Panstrongylus*, *P. megistus* (Carcavallo et al. 1998 - without detail) and *P. geniculatus* (Garrouste 2009 - fed on Lepidoptera haemolymph) are reported to practice haemolymphagy. Additional studies are required to understand the importance and type (intraspecies, intrasubfamily or extrasubfamily) of haemolymphagy for *P. rufotuberculatus*. Finally, *R. stali* is an Amazonian species living in palm trees and potentially feeding on birds and mammals (Carcavallo et al. 1998). In the Pantanal region of Brazil, *R. stali* is reported to live in arboreal nests of coatis (de Lima et al. 2015). In Bolivia, they are also found in the peridomiciles of human dwellings, especially in association with hens, and some domestic infestations have been reported (Matias et al. 2003, Justi et al. 2010, Martínez et al. 2012). They were found together with various species of cockroaches (Depickère, pers. com). Nevertheless, under our conditions, neither the N1, nor the adults, fed on cockroaches. Their resistance to starvation was higher than that of *P. rufotuberculatus* but lower than that of *T. infestans*. In the genus *Rhodnius*, *R. prolixus* was revealed to practice intraspecies cleptohaematophagy (Marinkelle 1965); haemolymphagy was not reported.

For the 37 N1 of *E. mucronatus*, only one survived to the adult stage. This result suggests that survival in this species is compromised by a diet of cockroach haemolymph alone. On the other hand, it also suggests that haemolymph provides sufficient nutriment to allow development into a fertile adult. Compared to a mouse diet, the cockroach diet seemed to increase (1) the mortality at each instar, (2) the number of feedings needed to molt, (3) the volume of maximum food intake, and (4) the time needed to molt. This species lives in Bolivia in peridomiciles, where they were found together with cockroaches (Depickère et al. 2012). In the wild, *E. mucronatus* lives in large, hollow trees; adults feed on porcupines (*Coendou prehensilis*), and the youngest instars have been observed feeding on the haemolymph of the large arachnids (*Amblypygi*) that inhabit hollow trees (Miles et al. 1981, Carcavallo et al. 1998, Gaunt & Miles 2000). Our results suggest that this species could utilise the presence of cockroaches in their environment to facilitate survival. Haemolymph is probably not their principal host, but exploitation of this food source may be possible. Haemolymph facilitates survival at each instar, molting, and fertility.

*T. boliviana* is also able to exploit haemolymph from cockroaches to survive. Nevertheless, they have more challenges in exploiting this food source than *E. mucronatus*. Indeed, their mortality rate was very high, especially for N1. With just a cockroach diet, none of the N1 developed into adult in this experiment. The effects of the cockroach diet were similar to those observed in *E. mucronatus*, including an increase in the mortality per instar, number of feedings needed to molt, volume of food intake, and time needed to molt. The provision of a choice between a mouse and a cockroach did not seem to improve development, especially for N1 instars, when compared with a cockroach diet. This result was not found for N2 (CcChCc diet), for which biological development was closer to that of the N2 reared on a mouse diet. This could suggest a difficulty for the N1 nymphs in finding the mouse that was at the top of the container. On the contrary, N2 utilise both food sources and were stronger and had superior motility. Although they were able to feed on haemolymph, our data suggest that the N2 and N3 nymphal instars preferred feeding on mice.

Generally, haemolymphagy is predominately cited as a survival practice for the youngest instars. We hypothesised similar behaviour in *T. boliviana*, in which N1 mortality was very high when reared under laboratory conditions with mice as a food source (Durán et al. 2014). When the cockroach and mouse diets were compared, there was no difference in the number of N1 that died without feeding or the age at first meal. Hence, the attraction towards the cockroach was similar to that towards the mouse. The highest mortality in N1 instars was observed in cases in which nymphs fed and died before molting; this was observed in both *T. boliviana* and *E. mucronatus* species. These nymphs were characterised by a low median weight increase. Two hypotheses can be put forward: (i) access to the cockroach haemolymph presents an additional challenge and/or (ii) the haemolymph nutriments are not an optimal food source for triatomines. Some observations supported the first hypothesis; in the experiments, the cockroaches used as a food source were adults or N5. They were of greater size (5-8 cm) than the triatomine N1 instars (3 mm). On a few occasions, the N1 individuals were found dead, squashed by the cockroach. Therefore, the adult cockroach might represent a danger for the youngest triatomine instars, and this could decrease the success of feeding on haemolymph, even if the triatomine saliva is known to have a paralysing effect on the host (Alves et al. 2011). The youngest cockroach instars may be better prey. On the other hand, some other observations support the second hypothesis: the youngest instars needed to feed at a greater quantity and frequency to molt. Even as adults, they fed more often or had a higher volume of intake on the cockroach diet when compared with the mouse diet. This suggests that haemolymph may be digested more quickly than blood, and so represented a challenge in developing to the next instar.

Interestingly, the lifespan of the single male of *E. mucronatus* and the single male of *T. boliviana* that completed their development was relatively long. No statistical data can be expressed using only data from two individuals, but, for example, adults of *B. ferroae* have been suggested to have a longer lifespan on a cockroach diet relative to a mouse diet (Sandoval et al. 2013). Previous studies have suggested that the blood diet is deficient in several essential factors, such as vitamin B (Lehane 2005), and the digestion of haemoglobin results in the production of large amounts of haeme, a potentially cytotoxic molecule that can exert biological damage (Graça-Souza et al. 2006, Donohue et al. 2009). On the other hand, the female *T. boliviana* died relatively quickly; that may simply be an unfortunate case or it may suggest that the import of blood is more important for females. Further studies are needed to investigate these issues.

Finally, as explained previously, *E. mucronatus* and *T. boliviana* in Bolivia have a very low infection index, 0% in *T. boliviana* (microscopy) and 0% (microscopy) and 19% (PCR) in *E. mucronatus* (Noireau et al. 1995, Depickère et al. 2012)*.* The hypothesis that a diet based on haemolymph (cockroach haemolymph, in our case) could reduce triatomine infection was not supported by our results, with parasites being still easily observed in the faeces of the experimental insects by microscopy after 70 days. Further experiments should be carried out to test the effect on the *T. cruzi* strain of a haemolymph food source.

In conclusion, *T. boliviana* and *E. mucronatus* are able to feed on the haemolymph of cockroaches, which could be a timely food source to improve the survival rates. The results of this study represent a first step in increasing knowledge of the effect of diets on the biological cycle of triatomines and suggest additional questions. A field study of triatomine meal sources is strongly recommended to better characterise their alimentary habits and to observe the real rate of feeding on cockroaches or other arthropods in the field. The degree of cleptohaematophagy, intraspecific haemolymphagy and phytophagy should also be investigated.
